# Workplace Violence and Harassment Against Emergency Medicine Residents

**DOI:** 10.5811/westjem.2016.6.30446

**Published:** 2016-07-19

**Authors:** Benjamin H. Schnapp, Benjamin H. Slovis, Anar D. Shah, Abra L. Fant, Michael A. Gisondi, Kaushal H. Shah, Christie A. Lech

**Affiliations:** * Northwestern University, Feinberg School of Medicine, Department of Emergency Medicine, Chicago, Illinois; † Mount Sinai Hospital, Icahn School of Medicine, Department of Emergency Medicine, New York, New York; ‡ Columbia University, Department of Biomedical Informatics, New York, New York; § New York University School of Medicine, Bellevue Hospital Center, NYU Langone Medical Center, Department of Emergency Medicine, New York, New York

## Abstract

**Introduction:**

Several studies have shown that workplace violence in the emergency department (ED) is common. Residents may be among the most vulnerable staff, as they have the least experience with these volatile encounters. The goal for this study was to quantify and describe acts of violence against emergency medicine (EM) residents by patients and visitors and to identify perceived barriers to safety.

**Methods:**

This cross-sectional survey study queried EM residents at multiple New York City hospitals. The primary outcome was the incidence of violence experienced by residents while working in the ED. The secondary outcomes were the subtypes of violence experienced by residents, as well as the perceived barriers to safety while at work.

**Results:**

A majority of residents (66%, 78/119) reported experiencing at least one act of physical violence during an ED shift. Nearly all residents (97%, 115/119) experienced verbal harassment, 78% (93/119) had experienced verbal threats, and 52% (62/119) reported sexual harassment. Almost a quarter of residents felt safe “Occasionally,” “Seldom” or “Never” while at work. Patient-based factors most commonly cited as contributory to violence included substance use and psychiatric disease.

**Conclusion:**

Self-reported violence against EM residents appears to be a significant problem. Incidence of violence and patient risk factors are similar to what has been found previously for other ED staff. Understanding the prevalence of workplace violence as well as the related systems, environmental, and patient-based factors is essential for future prevention efforts.

## INTRODUCTION

Nearly two million assaults occur annually as a result of workplace violence in the United States, with 12% occurring in the healthcare industry.^1^ Workplace violence is defined as any act or threat of physical violence, harassment, intimidation, or other threatening and disruptive behavior at one’s place of employment.^2^ One of the highest risk areas in the hospital for workplace violence is the emergency department (ED).^3^ Staff members care for acutely ill and injured patients with a broad spectrum of undifferentiated medical conditions, social issues, and psychiatric disease that may cause these individuals to become unpredictably aggressive or violent during their stay.

In a recent survey of ED staff, 51% of physicians reported being physically assaulted by a patient or visitor.^4^ In 20% of EDs, guns or knives are present daily or weekly,^5^ and the ED is the most frequent site for hospital shootings.^6^ Even verbal threats can be a serious problem, as they have been shown to increase the risk of future serious incidents of violence.^7^ Despite these risks, 50% of residents feel that their hospital security is inadequate.^8^ To our knowledge, there has been only one previous study that focused exclusively on the resident experience with physician harassment, where McNamara et al demonstrated nearly universal harassment (98%) with an associated increased risk of physician burnout.^9^ For over 20 years, there has been no new research that focuses exclusively on violence against residents in the ED.

Physicians with fewer years of training have been shown to be more often subjected to workplace violence,^10^ and are likely to have less experience and training in managing such difficult situations. Residents may also be less inclined to document violent events for fear of consequences, and are known to experience significant barriers to reporting these sentinel events.^11^ The purpose of this study was to quantify and describe the current incidence of violence and harassment against emergency medicine (EM) residents by patients and visitors, as well as identify current perceived barriers to safety in the ED.

## METHODS

### Study Design, Setting, and Population

This was a cross-sectional survey study conducted at three EM residency training programs based within the Mount Sinai Health System in New York City, including four large tertiary care facilities as well as two public hospitals and two trauma centers with a combined census of over 500,000 annual ED visits. All 142 EM residents within the Mount Sinai system were eligible to participate (60 at one postgraduate year [PGY]1–4 program and 82 at two PGY1–3 programs).

This study was approved as exempt research by the institutional review boards at each of the participating institutions.

### Study Protocol

Two independent investigators searched PubMed using the search terms “violence,” “assault,” “emergency department,” “staff,” “residents,” “housestaff,” and “health care workers” to identify a potential survey instrument. Among the studies identified through this search, the investigators determined by mutual agreement which set of survey questions had the greatest applicability to the resident population to be evaluated. The survey instrument selected as most relevant was published by Gates et al (2006) and included a variety of multiple-choice questions regarding the amount and types of violence (e.g. physical, sexual) that staff experienced or witnessed as well as perceived barriers to safety in their EDs (see Appendix A).

The residency leadership team at Mt. Sinai, comprised of the program director, assistant program directors and chief residents, reviewed the survey instrument and adapted it. Questions unrelated to the experience of resident physicians were removed from the original Gates instrument. Additionally, exhaustive definitions of each type of violence that were included in the original study were omitted and condensed descriptors were substituted for brevity; language limiting the reportable incidents to the previous six months was also removed as off-service rotations creating uneven amounts of time in the ED was thought to be a potential confounder. The revised instrument was piloted on two recent Mount Sinai Health System residency graduates who were not a part of the study; slight revisions were made to the wording of questions for clarification.

Paper copies of the survey instrument were distributed to residents at the time of their 2015 annual American Board of Emergency Medicine In-Training Examination (ABEM ITE). Participation in the study was optional, and responses were kept anonymous. Residents were informed that exclusion or non-participation in the study would not affect their performance on the ABEM ITE or their resident standing, and instructed to report their personal experiences only. Completion of the survey implied assent. We excluded from participation residents who were absent from the ABEM ITE or did not complete the survey at that time. Surveys were collected at the end of the ABEM ITE, and results were entered into a secure database.

To compare respondent demographics to those of the entire population in order to assess for nonresponse bias, we reviewed demographic data provided by each residency. Sex demographics of the entire population were compared to those that anonymously participated in the survey.

### Outcome Measures

The primary outcome was the incidence of violence experienced by residents while working in the ED during their EM residency thus far. The secondary outcomes were the subtypes of violence experienced by residents as well as the perceived barriers to safety while at work.

### Data Analysis

Completed survey data were entered into a secure Microsoft Excel (2011, Redmond WA) spreadsheet by a trained research assistant blinded to the objectives of the study. We calculated descriptive statistics as well as one-way ANOVA and odds ratios using IBM SPSS version 20.0 (2011, Armonk NY).

## RESULTS

### Demographics

The response rate to our survey was 84% (119/142). Female residents comprised 37.8% (44/119) of the participants. This was highly representative of the overall population based on review of the residency websites, which demonstrated a female population of 37.3% (52/142). There were 36 PGY1, 35 PGY2, 36 PGY3, and 12 PGY4 residents across all residencies of the 119 residents who completed the survey. Mount Sinai represented 52 of the 119 respondents, Mount Sinai Beth Israel represented 39 of the 119 respondents, and Mount Sinai Saint Luke’s Roosevelt represented 28 of the 119 respondents.

### Physical Abuse

A majority of subjects (65.5%, 78/119) reported an experience of physical violence in the ED committed by a patient, while 11.8% (14/119) reported experiencing violence committed by visitors. The median number of times a resident experienced violence by a patient during their EM residency was 1.0 (IQR [2.0–0.0]) while the median number of times a resident experienced violence from a visitor was 0.0 (IQR [0.0-0.0]). The frequency of number of reported violent incidents committed by patients and visitors is displayed in Table 1.

There was a statistically significant difference between PGY level and frequency of violent incidents from patients as determined by one-way ANOVA F(3,115)=5.3, p=0.002. A Tukey post-hoc analysis revealed a statistically significant difference in violent incidents between PGY1s (0.7 ± 1.2) when compared to PGY2s (1.9±2.1, p=0.02 CI [0.13–2.3]) and PGY3s (2.2±1.6, p=0.002 CI [0.42–2.5]), however no significant difference when compared to PGY4s (2.0±2.1, p=0.105 CI [−0.2–2.8]). There was no significant difference for PGY2s when compared to PGY3s (p=0.899 CI [−1.3–0.77]) or PGY4s (p=0.997 CI [−1.4–1.6]), and PGY’s had no statistically significant difference in attacks when compared to PGY3’s (p=0.991 CI [−1.6–1.3]), suggesting both a consistent pattern of exposure time and violence and no protective or harmful effect of years of experience. Mean number of violent incidents for each PGY level is depicted in the Figure.

### Verbal Harassment

Nearly all of the respondents (115/119, 96.6%) reported prior verbal harassment from a patient in the ED. Slightly fewer (103/119, 86.6%) reported prior verbal harassment from a visitor. More than three quarters (93/119, 78.2%) reported having experienced verbal threats from a patient while a majority (66/119, 55.5%) reported prior verbal threats by a visitor. Further descriptive statistics can be found in Table 2.

### Sexual Harassment

Sexual harassment by a patient was reported by a majority (62/119, 52.1%) of the residents. Of these, 31/74 (41.9%) of the male responders reported sexual harassment, while 31/45 (68.9%) of the female responders reported the same. Descriptive statistics for sexual harassment are provided in Table 2. There was an increased odds ratio between gender and likelihood of sexual harassment (OR=3.071 95% CI [1.4–6.7]). Sexual harassment by a visitor was reported by 26/119 (21.8%) of the respondents, with 15/74 (20.3%) of the male and 11/45 (24.4%) of the females reporting this experience. There was minimal difference in odds between these groups with OR=1.273 (95% CI [0.5–3.1]). Resident reported sexual harassment is further characterized in Table 2.

### Contributing Factors

Almost all of the respondents (118/119, 99.2%) reported that certain patient factors contribute to physical abuse. Among these, the most frequently reported were alcohol (113/119, 95.0%) and drug use (112/119, 94.1%). Psychiatric disease was also reported frequently as a contributing factor with (109/119, 91.6%) respondents reporting that this may have contributed to their experiences, while 70/119 (58.8%) reported organic causes such as dementia leading to physical abuse. The patient’s inability to deal with a crisis situation was cited by 76/119 (63.9%) of the respondents cited as a factor for physical abuse.

Nearly all participants (118/119, 99.2%) reported environmental factors such as a lack of security or police presence (82.4%; 95% CI [75.5–89.2%]), and security or police not responding in a timely manner (68.1%; 95% CI [59.7–76.4%]) as the most common contributors to physical abuse. Patient areas being open to the public, and ease of bringing weapons into the ED were both cited by 69 (58.0%) as leading to increased risk of violence.

Slightly fewer residents (115/119, 96.6%) reported staffing factors as a cause of physical abuse, with a lack of adequate staff (79.8%; 95% CI [72.6–87.0]) being the most common. Slightly less than half (59/119, 49.6%; 95% CI [40.6–58.6%]) of respondents felt that working evening and nights made them more likely to encounter violence.

### Workplace Safety

Nearly a quarter of resident responders (27/119, 22.7%) reported feeling “Occasionally,” “Seldom” or “Never” safe while at work in the ED. Almost half (58/119, 48.7%) felt “Very Dissatisfied” or “Somewhat Dissatisfied” with the current security in their ED. Tables 3 and 4 demonstrate the descriptive statistics for questions regarding workplace safety in our survey.

### Violence Prevention

A small minority of residents (20/119, 16.8%) confirmed prior training in violence prevention or de-escalation techniques, 17 of these within the preceding 12 months. Nine of these (45%) were PGY 1s, with four (20%), three (15%) and four (20%) representing PGY 2, 3 and 4s respectively. Fourteen (74%) of those who took violence prevention training courses were male.

## DISCUSSION

In the past two decades, only one other study has focused exclusively on the resident physician experience with violence in an American urban ED, with our results demonstrating similar measurements of violence against emergency physicians in training. Our response rate was overall high, with 84% of potential respondents participating in the study. Respondent demographics were comparable to the overall population of the participating residencies (62.6% male, 37.3% female) and also appear to be representative of the demographics of emergency medicine across the country, with the latest documentation from the Association of American Medical Colleges demonstrating EM residents as being 63% male and 37% female.^12^

Our results demonstrate that 78% of respondents experienced at least one act of workplace violence, which is consistent with a previous study reporting a rate of 76%,^13^ but higher than another report that 37% of resident physicians had experienced physical violence committed by patients and visitors.^9^ Touzet et al reported an even higher rate of violence committed against ED staff, with 96% experiencing a violent incident (although this study population also included nurses and medical technicians).^3^ Only 16% of residents in our study reported prior training in violence prevention or de-escalation techniques. This percentage is similar to prior studies in which 14% of ED physicians overall had previously participated in violence training/workshops.^14^

The patient-based factors reported in this study to correlate most highly with propensity to violence (alcohol, drug use, and history of psychiatric disease, 95%, 94.1%, and 91.6%, respectively), are similar to what has been reported in prior literature.^3^,^8^ Previous studies of ED-based workplace violence have found that 84% of all physician respondents believed that patients making verbal threats were intoxicated “frequently or most of the time.” Similarly, 68% of physicians believed that those patients who had physically assaulted them in the ED were intoxicated “frequently or most of the time.”^8^

The majority of residents in this study reported at least one incidence of verbal harassment (96.6%) or verbal threats (78.2%). This is higher than previously reported levels of verbal harassment of residents by patients and visitors of 86.1% and verbal threats of 60.9%.^9^ Over half (52.2%) of residents in the current study reported being sexually harassed by a patient, and the majority also reported sexual harassment (68.9% of female participants, 41.9% of male participants). These findings suggest a higher prevalence of sexual harassment, especially of male residents, than previously reported.^3^

While it is important to note that 77.3% of the residents in our study reported feeling safe at work “Often” or “Always,” nearly a quarter of residents felt safe “Occasionally,” “Seldom” or “Never” while working in the ED. These results are nearly identical to prior studies in which 25% of all eemergency physician respondents reported feeling safe at work “Sometimes,” “Rarely,” or “Never.”^4^,^8^ A study of all ED staff found that only 7.2% “always” felt safe in the ED, similar to the 10.9% of residents who “always” felt safe in our study. An unsafe work environment has been shown to correlate with negative mental health effects,^15^ increased costs to the hospital,^16^ decreased productivity at work,^17^ and decreased job satisfaction for ED staff, along with having harmful effects on patient care.^18^ A work environment that is perceived as unsafe also detracts from resident wellness: residents questioned their decision to become emergency physicians, and experienced emotional and family disruption as a result of workplace violence.^9^ Although our study found similar rates of violence against residents as previous studies that included a broader range of workers in the ED, it is interesting to note that residents, attending physicians, nurses and other ED staff each have very different levels and rates of contact with potentially violent patients.

Self-reported violence against EM resident staff committed by patients and visitors is a significant concern within the ED environment, and the majority of residents in this study reported being the victim of at least one incident of physical violence or sexual harassment in the ED. If these results are validated with prospective, observational data, ED security policies and staffing should be examined to ensure that they are maximizing the safety of ED staff. On an institutional level, hospital leadership can commit to a comprehensive violence reduction plan.^7^ On a state or national level, policy changes that clearly define a no-tolerance policy for ED violence would likely help reduce the incidence of violence against all ED staff. As our study found a higher rate of sexual harassment for physicians than has been previously reported, future research could aim to confirm these findings and examine specific interventions to target this behavior. Additionally, nonviolence and de-escalation techniques should be further investigated as a possible method to reduce violent incidents in the ED.^19^ If successful, these training programs could be explored as a possible addition to the ABEM Model of Clinical Practice for Emergency Medicine.

## LIMITATIONS

The study was based on self-reported survey responses, so the results may be vulnerable to recall bias; as with all surveys, there is no way to objectively verify the accuracy of the reported incidents. This was a cross-sectional study and therefore represents only one moment in time. The time of year of the survey, as well as several well-publicized incidents of violence in the depertment in the months before the survey may have influenced the residents’ recall. This study asked residents at all stages of their training to report their exposure to violence without controlling for the number of months spent in the ED (due to PGY year or off-service rotations), which may have affected reported levels of violence. This survey was provided to residents at urban, academic hospital centers and may not be representative of other hospital or clinical settings with a different patient populations and less alcohol/substance abuse or psychiatric disease. However, given the number and variety of New York hospitals surveyed, the results are likely representative of the experience in the city at large. The response rate for this study was 84%. We were unable to account for demographic information for those residents who did not participate in the survey, as we did not collect data from this group. However, we did attempt to compare the demographics of those who completed the survey with the residency populations as a whole based on each residency’s respective website. While participation holds the potential for selection bias, we presume a lack of participation was most likely due to missing the ABEM ITE. Missing the ABEM ITE was most likely due to clinical responsibilities or vacation; therefore, those who did not respond to our survey were unlikely to be systematically different from our respondents, and our analysis of the demographics of the residency websites seems to confirm this. It is possible that residents who responded to the survey may have had experienced a greater or fewer number of episodes of violence than the population at large, but we consider systematic bias unlikely. While we attempted to include all of the most commonly cited reasons for ED violence in our survey, it is possible there were other reasons missed by our instrument. Additionally, our modified survey instrument has not been validated for use in the ED population, though modifications for our study were minimal.

## CONCLUSION

In summary, workplace violence is experienced by New York City residents at an alarming rate, similar to previously reported rates of violence for attending physicians and other ED staff. Psychiatric disease and substance use among patients are reported risk factors for increased threats and violence. As a workplace that is perceived to be unsafe has been shown in other studies to affect job satisfaction and patient care, further research is warranted to determine the parameters that affect violence in the ED.

## Supplementary Information



## Figures and Tables

**Figure:**
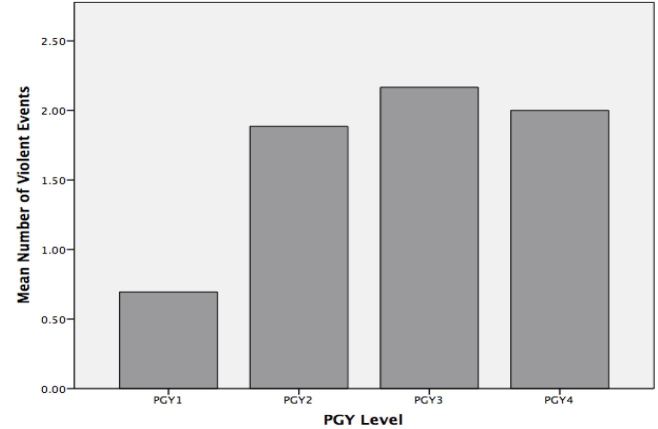
Mean number of violent events from patients by postgraduate year (PGY) level.

**Table 1:** Number of violent incidents reported by residents, with the second column representing events committed by patients, and the third representing those committed by visitors.

Patients			Visitors	
	
Incidents	Frequency	Percent	Frequency	Percent
	
0	41	34.5	105	88.2
1	26	21.8	9	7.6
2	25	21.0	4	3.4
3	11	9.2	1	0
4	7	5.9	0	0
5	3	2.5	0	0
6	1	0.8	0	0
7+	5	4.2	0	0
Total	119	100	119	100

**Table 2:** Descriptive statistics for those residents who experienced verbal harassment, verbal threats and sexual harassment in the emergency department from patients and visitors.

Report		Frequency	Percent
	
Verbal harassment	From patient (n=119)	115	96.6
	From visitor (n=119)	103	86.6
Verbal threats	From patient (n=119)	93	78.2
	From visitor (n=118)	66	55.9
Sexual harassment	From patient (n=119)	62	52.1
	From visitor (n=119)	26	21.8

**Table 3:** Frequency at which residents reported feeling safe at work.

	Frequency	Percent
Never	8	6.7
Seldom	1	0.8
Occasionally	18	15.1
Often	79	66.4
Always	13	10.9
Total	119	100.0

**Table 4:** Resident satisfaction with emergency department security*.*

	Frequency	Percent
Very dissatisfied	16	13.4
Somewhat dissatisfied	42	35.3
Neutral	22	18.5
Somewhat satisfied	29	24.4
Very satisfied	10	8.4
Total	119	100.0
