# Supplementary material for: Workplace Violence and Harassment Against Emergency Medicine Residents
Source: West J Emerg Med. 2016 Jul 19;17(5):567–73. doi: 10.5811/westjem.2016.6.30446 (PMC5017841; doi:10.5811/westjem.2016.6.30446)
Supplement: Supplementary file 1 [file wjem-17-567-s001.docx]

**Appendix A**

**EMERGENCY DEPARTMENT VIOLENCE/SAFETY SURVEY**

**Demographics**

I) What is your gender

1. Male
2. Female

II) What is your position/PGY year?

III) What is/are the hospital site(s) at which you rotate/work:

1. Mount Sinai Hospital Center
2. Elmhurst Hospital Center
3. Beth Israel Hospital
4. St. Luke’s Hospital
5. St. Luke’s Roosevelt Hospital
6. Mount Sinai Queens
7. Other, please specify:

**Non-Physical Violence Experiences**

IV) Have you ever experienced verbal harassment (i.e. cursing, yelling, racial slurs, humiliating actions) from a patient?
a. Yes

b. No

V) Have you ever experienced verbal harassment (i.e. cursing, yelling, racial slurs, humiliating actions) from a visitor?
a. Yes

b. No

VI) Have you ever experienced verbal threats (verbal threats/body language/written threats expressed with intent to harm) from a patient?
a. Yes

b. No

VII) Have you ever experienced verbal threats (verbal threats/body language/written threats expressed with intent to harm) from a visitor?
a. Yes

b. No

VIII) Have you ever experienced sexual harassment (unwelcome sexual advances, insulting gestures, requests for sexual favors, offensive contact) from a patient?
a. Yes

b. No

IX) Have you ever experienced sexual harassment (unwelcome sexual advances, insulting gestures, requests for sexual favors, offensive contact) from a visitor?
a. Yes

b. No

**Physical Violence Experiences**

X) How many times have you been physically attacked (hitting slapping, punching, kicking, spitting, shooting, stabbing, biting, hitting with an object, throwing an object) by a patient?
a) Never

b) Once

c) Twice

d) Three times

e) Four times

f)  Five times

g) Six times

h) Seven or more times

XI) How many times have you been physically attacked (hitting slapping, punching, kicking, spitting, shooting, stabbing, biting, hitting with an object, throwing an object) by a visitor?
a) Never

b) Once

c) Twice

d) Three times

e) Four times

f)  Five times

g) Six times

h) Seven or more times

Contributing Factors to ED Violence

XII) Please check off all the factors that below that you believe are patient and visitor factors that contribute to physical assaults:

_____ Alcohol use

_____ Drug use

_____ Psychiatric disease

_____ Organic brain syndrome/dementia

_____ Inability to deal with crisis situation

_____ Gang involvement

XIII) Please check off all the staffing factors that contribute to physical assaults:

_____ Lack of adequate staff

_____ Evening/night shifts

_____ Lack of information about patients/visitors with prior violence history

_____ Being alone with a patient or visitor

_____ Lack of violence prevention training

_____ Working long hours

XIV) Please check off all the environmental/hospital-based factors that below that you believe contribute to physical assaults:

_____ Long wait time for patients

_____ Lack of security/police presence

_____ Patient areas/triage open to public

_____ Security/police do not respond in a timely manner when called

_____ Easy of ability to bring weapons into the ED

_____ Lack of metal detectors/alarms

_____ Security are present but not helpful

_____ Lack of policies/procedures for handling known violent offenders

**Prior Violence Prevention Training**

XV) Have you previously receiving any form of violence prevention training in the past 12 months?

1. Yes
2. No

XVI) If you replied YES to question XV, did you receive violence prevention training within the last 12 months with your current employer?

1. Yes
2. No

XVII) If you replied YES to question XV, did you receive violence prevention training within the last 12 months from somewhere besides your current employer?

1. Yes
2. No

**Job Safety and Satisfaction**

XVIII) How often do you feel safe (free from violence) while working in the emergency department?

1. Never
2. Seldom
3. Occasionally
4. Often
5. Always

XIX) How satisfied are you with your current position/job title?

1. Very dissatisfied
2. Somewhat dissatisfied
3. Neutral
4. Somewhat satisfied
5. Very satisfied

XX) How satisfied are you with the emergency department?

1. Very dissatisfied
2. Somewhat dissatisfied
3. Neutral
4. Somewhat satisfied
5. Very satisfied

XXI) How satisfied are you with your hospital(s)?

1. Very dissatisfied
2. Somewhat dissatisfied
3. Neutral
4. Somewhat satisfied
5. Very satisfied

XXII) How satisfied are you with the security in your emergency department?
a. Very dissatisfied

b. Somewhat dissatisfied

c. Neutral

d. Somewhat satisfied

e. Very satisfied

XXIII) What would make you feel safer while working in the ED?
